# Supplementary material for: In situ synthesis and dynamic simulation of molecularly imprinted polymeric nanoparticles on a micro-reactor system
Source: Nat Commun. 2023 Aug 10;14:4840. doi: 10.1038/s41467-023-40413-8 (PMC10415298; doi:10.1038/s41467-023-40413-8)
Supplement: Supplementary file 1 — Supplementary Information [file 41467_2023_40413_MOESM1_ESM.pdf]

Supplementary Information

In situ Synthesis and Dynamic Simulation of Molecularly Imprinted Polymeric Nanoparticles on a Micro-Reactor System

Özgecan Erdem[1], Ismail Eş[1], Yeşeren Saylan[2], Maryam Atabay[1,2], Murat Alp Gungen[1,3], Kadriye Ölmez[1], Adil Denizli[2], Fatih Inci[1,3,*]

[1] *UNAM-National Nanotechnology Research Center, Bilkent University, 06800, Ankara, Turkey*

[2] *Department of Chemistry, Hacettepe University, Ankara, 06800, Turkey*

[3] *Institute of Materials Science and Nanotechnology, Bilkent University, 06800, Ankara, Turkey*

*Corresponding author: finci@bilkent.edu.tr

1. Supplementary Materials and Methods

1.1. Materials

Poly (vinyl alcohol) (PVA), sodium dodecyl sulfate (SDS), sodium bicarbonate ($NaHCO_3$), 2-hydroxyethyl methacrylate (HEMA), ethylene glycol dimethacrylate (EGDMA), methacrylic acid (MA), ammonium per sulfate (APS), sodium bisulfite ($NaHSO_3$) and poly-L-lysine (PLL), sodium hydroxide (NaOH), sodium chloride (NaCl), bovine serum albumin (BSA) and acetic acid was purchased from Sigma-Aldrich (MO, USA). Phosphate-buffered saline (PBS) was purchased from Gibco. The Sylgard™ 184 Silicone Elastomer Kit was obtained from Dow Corning (Auburn, MI, United States) for the production of positive molds. Isopropyl alcohol was purchased from Isolab Laborgeräte GmbH (≥99.5%, Eschau, Germany). Poly (methyl methacrylate) (PMMA) was purchased from Sumitomo Chemical (Singapore), double-sided adhesive (DSA) films were obtained from 3M (Saint Paul, MI, United States) and PTFE tubings were purchased from Adtech (Gloucestershire, England) to fabricate micro-reactors.

1.2. Computational Analyses

1.2.1. Preparation of structures

We initially used the sdf files of MA and HEMA published on PubChem website [4,5]. Then, these files were converted to PDB files by using USCF Chimera 1.16 software [6]. The PDB structure of MA-HEMA was made on GaussView 6.0 [7]. After preparing three PDB files, we completed the input files on CHARMM-GUI website [8]. Next, these structures were optimized at the DFT level using Gaussian09 [9] by employing B3LYP hybrid functional [10] and the 6-31G (d) basis set. Finally, the chain B of BSA (PDB ID: 4f5s) was used for all the simulations.

1.2.2. Docking studies

In order to evaluate the most favorable positions on protein structure, we performed molecular docking studies between (i) MA and BSA and between (ii) MA-HEMA dimer and BSA. In this regard, we used three platforms i.e., HDOCK (http://hdock.phys.hust.edu.cn/) [11], Swissdock (http://www.swissdock.ch/) [12], and Autodock Vina in UCSF Chimera [6], and the results of all methods confirmed each other.

1.2.3. Molecular dynamics simulations

Molecular dynamics (MD) simulations were performed by GPU enabled version of NAMD [13]. All visualizations and analyses were carried out by VMD [14]. In the simulations, we considered three different scenarios. We assessed (1) the complex of BSA-1 molecule of MA with 10 molecules of HEMA molecules around and (2) the complex of BSA-2 molecules of MA with 20 molecules of HEMA molecules around and in water box with the dimensions of $123 \times 93 \times 105$ Å^3 for both scenarios. Here, each HEMA molecule was arranged in 8 Å from the surface of BSA randomly (in the distance less than cut-off distance). Approximated number of MA and HEMA were selected according to their molar ratio in the experiment (45×10^{-5} mol/L for MA and 412×10^{-5} mol/L for HEMA). (3) We evaluated the complex of MA-HEMA and BSA in water box with the dimensions of $109 \times 91 \times 111$ Å^3. The intramolecular energies and intermolecular interactions between molecules were modeled by utilizing CHARMM36 force fields [15]. In addition, TIP3P water model [16] and 0.15 M of NaCl were used for solvation and neutralization, respectively. Periodic boundary conditions were applied for NPT ensemble by keeping temperature at 313 K and pressure at 1 bar using Langevin thermostat and Nose–Hoover Langevin piston, respectively. The evaluation of long-range columbic interaction was performed through Particle Mesh Ewald (PME) method [17], and a cut-off of 12 Å was set for the computation of short-range interactions. All scenarios were minimized in NVT and NPT ensembles respectively for 5000 steps to remove any steric clashes. Subsequently, all scenarios were equilibrated for 0.5 ns in the NPT ensemble. The production runs for each scenario were carried out with 1 fs time step and 20 ns number steps in the NPT ensembles.

1.3. Conventional synthesis of BSA-imprinted nanoparticles

Briefly, BSA-imprinted nanoparticles were synthesized with emulsion polymerization method, which consisted of two aqueous phases [1–3]. Briefly, the first phase included PVA (stabilizer) and SDS (surfactant) while the second aqueous phase contained PVA and SDS to form an emulsion and $NaHCO_3$ (pH adjuster). MA (functional monomer) and BSA (target molecule) were mixed for 1 h to generate a pre-complex. EGDMA (cross-linker) and HEMA (co-monomer) were mixed with the second solution, and then, homogenized at 24,000 rpm for 15 min to obtain micro-emulsions. After homogenization process, pre-complex was introduced to this solution and mixed with the

first aqueous phase. $NaHSO_3$ and APS were also added to the complete mixture as an initiator couple, and the polymerization was carried out at 40°C and 500 rpm for 24 h. After polymerization process, the resulting nanoparticles was centrifugated at 2415 x g for 5 min. Once the supernatant of these nanoparticles was used for further size and concentration analysis, the MIPs were washed with ethanol and water before desorption and further characterization studies. Washing with ethanol and water enables to remove impurities (for cleaning purposes only) and excess monomers, respectively. Moreover, the reason for using supernatant is to eliminate larger sizes of nanoparticles. The desorption of BSA protein from imprinted nanoparticles was carried out using 0.5 M of NaCl for an hour until no absorbance was observed at 280 nm. Non-imprinted polymers (NIPs) were prepared using the same protocol without using BSA.

Addition to these processes, we have tested the conventional production in 5, 10, 20, 30, 60, 90, 120, 150 and 180 min to compare them with the production results derived from the micro-reactor system. The collective dry-weight of MIPs per mL collected between 30-180 min intervals were also calculated after the MIPs were lyophilized.

1.4. Characterization of BSA-imprinted nanoparticles

1.4.1. Physicochemical analysis

Intensity-weighted mean hydrodynamic size (Z-average) and polydispersity (PDI) of BSA-imprinted nanoparticles were measured using dynamic light scattering (DLS, Zetasizer NanoZS, Malvern, UK). The backscattering configuration was set and the analysis was performed at a scattering angle of 173°. The He/Ne laser emission and power source to analyze BSA-imprinted nanoparticles were 633 nm and 4.0 mW, respectively. All samples were diluted in water (10:1) before the measurements.

In order to analyze the particle size, PDI, and concentration of BSA-imprinted nanoparticles, nanoparticle tracking analysis (NTA, Nanosight NS300, Malvern Instruments Ltd., Malvern, UK) was employed. Briefly, NTA employs a laser-based optical technique to monitor the Brownian motion of particles in solution supplied to the system. The system is coupled with an embedded 488 nm laser (blue) device, and the particles are analyzed by the instrument when their size is within the range of the optical properties of this laser [18]. Before initiating the analysis, distilled

water was passed through the system three times. All samples were diluted in distilled water to a final volume of 1 mL. Before the analysis, the ideal measurement concentrations were adjusted as there would be 20–100 particles/frame. For each sample, three video recordings with 60 sec of duration were taken and analyzed.

1.4.2. Chemical analysis

X-ray photoelectron spectroscopy (XPS, K-Alpha XPS, Thermo Fisher Scientific, USA) was performed in order to characterize MIPs and NIPs. Dried powder samples were placed to copper tapes and adjusted to the XPS table. Analysis was started after 30 min vacuum process. After surveying the general elemental map of the composition, all data were acquired from the software (Thermo Advantage).

1.5. Principal component analysis (PCA)

Principal component analysis (PCA) was applied to the data obtained from NTA to better assess the findings statistically and reduce the dimensionality of the data for a better interpretation. Pre-processing of the NTA data was performed on MatLab 2019. The data was first separated into two distinct groups. The first group involved the dataset obtained from the flow rates (labelled as F1 and F2). The second group was divided into three sub-groups based on the length of micro-reactor channels (Labelled as M1, M2, and M3). In both groups, the data was transformed into matrices where each column represented a nanoparticle size ranging between 1 nm and 1 μm, and each row represented an individual experiment. The orders of the matrices for the first and second groups were 53x1000 and 35x1000, respectively. In the first stage of preprocessing, the sizes of the matrices were reduced to 53x400 and 35x400 by cropping only the first 400 columns. The 0-400 nm interval was selected as the interval where the nanoparticle size distribution exists. In the second stage of preprocessing, the data was binned into three groups with the aim of focusing on the target nanoparticle size distribution. The first group included the average of the count of the particles smaller than the target size distribution (1-90 nm). The second group consisted of particles belonging to the target range (90-110 nm). The third and final group contained particles bigger than the target range (110-400 nm). After grouping, the data was transferred from MATLAB to GraphPad for the PCA.

1.6. Preparation of plasmonic metamaterial sensor

Firstly, with the purpose of obtaining a transparent surface, the coating layers on the optical disc were removed by physical and chemical processes, following the methods in previous study [19]. Finally, the transparent surfaces were coated with chromium (5 nm), silver (30 nm), and gold (15 nm) through an evaporation process to generate the plasmonic layers. Gold-coated surface was then incubated overnight in 0.05 mg/mL PLL solution to create amine terminated groups (Figure 6a). After incubation, PLL coated surface was washed with PBS. Then, the surfaces were assembled with poly (methyl methacrylate) (PMMA) (thickness: 2 mm) using a double side adhesive (DSA) layer (thickness: 50 μm), and inlet and outlet tubing were attached using an epoxy adhesive containing a resin and hardener (Pattex). Before binding studies of BSA, 1/10 diluted desorbed nanoparticle solution ($6x10^{12}$ particles/mL) was given to the sensor through a syringe pump (10 μL/min) for 20 min. After binding nanoparticles to the sensor surface, it was washed with distilled water for 10 min.

1.7. Characterization of plasmonic metamaterial sensor

The surface morphology of sensor and bare gold-coated nanoperiodic surface was characterized by atomic force microscopy (AFM, Asylum, Oxford Instrument, UK). The sensors PPP-NCHR tip was lowered into the sample until Z voltage value around 70-80 V, and the imaging process was applied with 0.6 Hz scanning rate. The images were analyzed using Gwyddion Software (ver 2.61).

1.8. Adsorption-desorption studies

After characterization studies, BSA-imprinted nanoparticles were tested with one adsorption-desorption cycle before using them in sensor. For this purpose, non-desorbed nanoparticles and 0.5 M NaCl solution (1:1) was shaken for 1 h at room temperature. Following that, nanoparticles were centrifugated for 45 min at 14.104 x g and supernatant was removed for absorbance measurements. Pellet was resuspended in water and then incubated with 1.5 mg/mL BSA for an hour in order to ensure that all bound target protein (BSA) were removed from the binding sites on the MIPs as demonstrated in the literature [20,21]. After centrifugation step, the supernatant was removed and measured at 280 nm. During this process, the target protein would not be denatured.

1.9. Kinetic studies

Before binding studies for BSA, sodium acetate buffer (pH 4) was adjusted to the system for 10 min to obtain a baseline. BSA solution (in pH 4) was then given to the system for 30 min. After the washing step for 10 min with the same buffer, BSA protein was desorbed with 0.5 M NaCl solution for 20 min and washed with water for 10 min. The flow rate of the pump (10 µL/min) and the time they were given to the system were the same in all experiments. Different concentrations of BSA solutions (10-50 µM) were interacted with BSA-imprinted plasmonic sensor. The real time responses (wavelength shifts) of the sensor were saved at the end of each analysis.

1.10. Selectivity, imprinting factor, and repeatability studies

The selectivity of BSA-imprinted nanoparticles was also examined. We herein applied 20 µM of human serum albumin (HSA) solution onto the plasmonic sensor, which was modified with BSA-imprinted nanoparticles, and we also compared the results derived from the same concentration of BSA applied to the same sensor. According to the results, the selectivity coefficient (k) was calculated. In addition, we calculated imprinting factor (relative selectivity coefficient (k')) by experimentally analyzing the binding of BSA proteins to the MIPs and NIPs. According to the results, selectivity and relative selectivity coefficients were also calculated. Likewise, a same concentration of BSA solution (30 µM) was interacted three times to test the repeatability performance of BSA-imprinted nanoparticles. Furthermore, 30 µM BSA solution were interacted with NIPs to observe and compare the responses with BSA-imprinted nanoparticles.

1.11. Statistical analysis

We statistically analyzed all the data using Violin-shaped Box–Whisker plots to evaluate the sizes of nanoparticles collected at 30-min intervals and then assessed with the nonparametric Freidman test followed by Uncorrected Dunn's multiple comparison test (Figure 2, Figure 4, Supplementary Figure 2 and 4).

2. Supplementary Figures

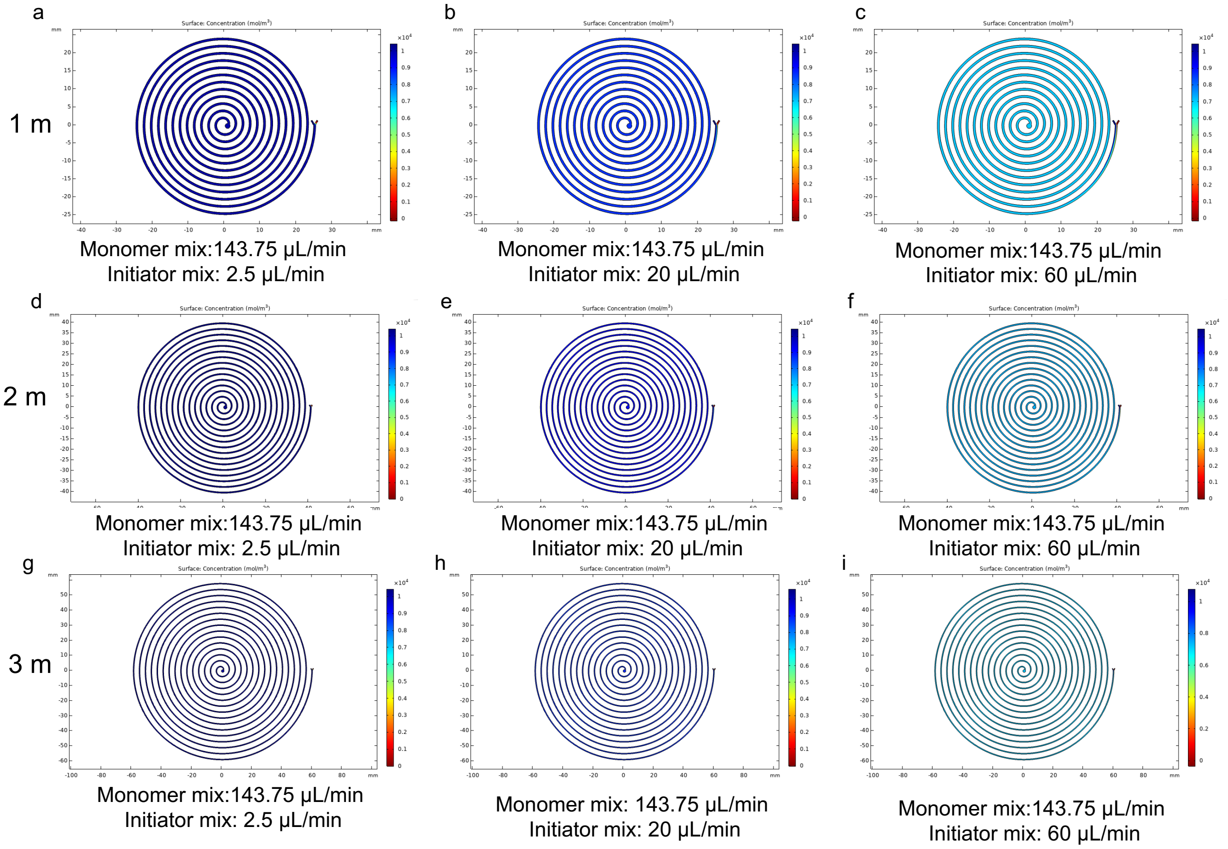

Supplementary Figure 1. COMSOL simulations for mixing efficiency. The combinations of flow rates applied in the micro-reactors, different lengths of (a-c) 1 m, (d-f) 2 m, and (g-i) 3 m are evaluated. The combinations of flow rates of (a, d, g) monomer mix include 143.75 µL/min, initiator mix: 2.5 µL/min; (b, e, h) monomer mix: 143.75 µL/min, initiator mix: 20 µL/min; and (c, f, i) monomer mix: 143.75 µL/min, initiator mix: 60 µL/min.

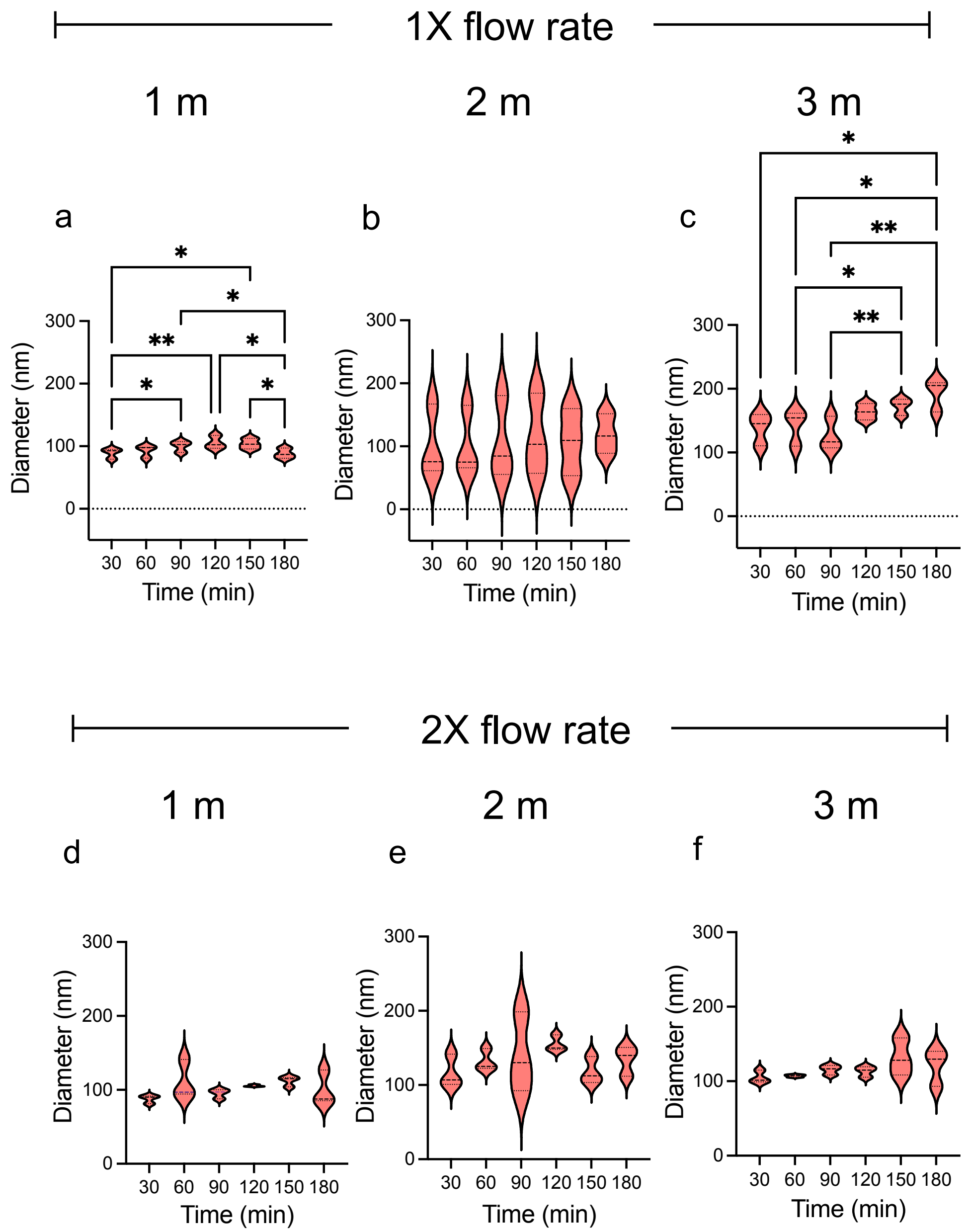

Supplementary Figure 2. DLS analysis. The DLS data of BSA-imprinted nanoparticles are shown for micro-reactors with different channel lengths and flow rates that include (a) 1m, (b) 2m, and (c) 3m at a flow rate of 1X, and (d) 1m, (e) 2m, and (f) 3m at a 2X flow rate. The data was analyzed by performing one-way ANOVA (Freidman test) statistical analysis, the statistical difference shown as *$p<0.05$ and **$p<0.01$.

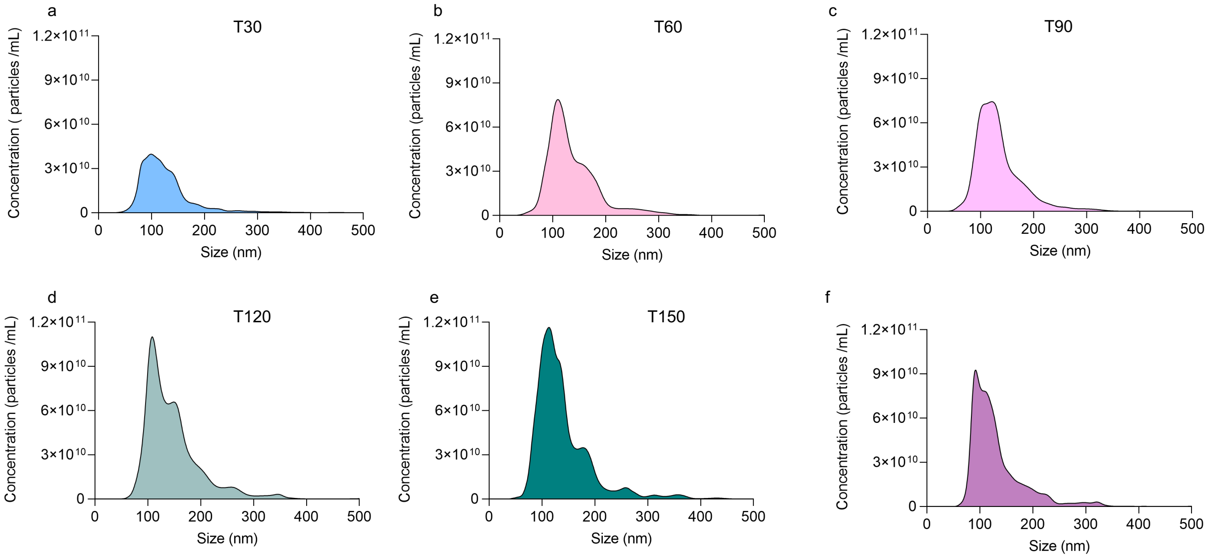

Supplementary Figure 3. The NTA analysis. (a-f) The data shows the concentration of nanoparticles collected at 30 min intervals over a period of 180 min, with a channel length of 1 m and a flow rate of 1X. The time intervals include (a) 30 min, (b) 60 min, (c) 90 min, (d) 120 min, (e) 150 min, and (f) 180 min.

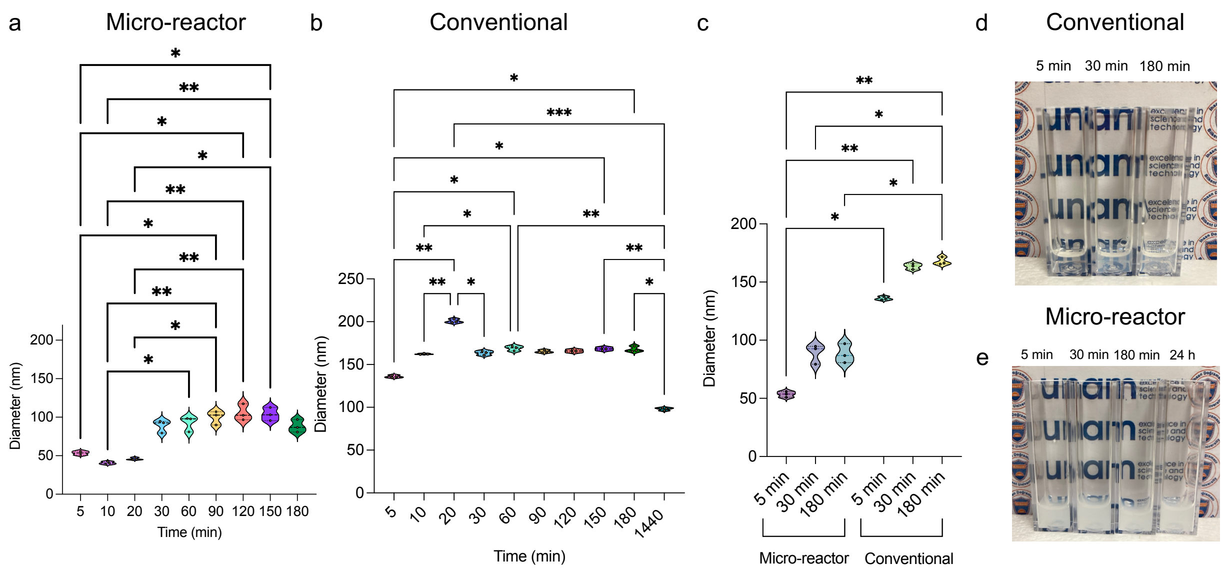

Supplementary Figure 4. DLS data and images of BSA-imprinted nanoparticles collected at time intervals varying from 0 to 180 min. DLS results are demonstrated for (a) micro-reactor and (b) conventional synthesized MIPs that are collected at different time intervals. (c) MIPs collected from micro-reactor and conventional method are compared after 5, 30, and 180 min. The photos of MIPs solutions synthesized on (d) micro-reactor and (e) conventional method are exhibited at these time slots.

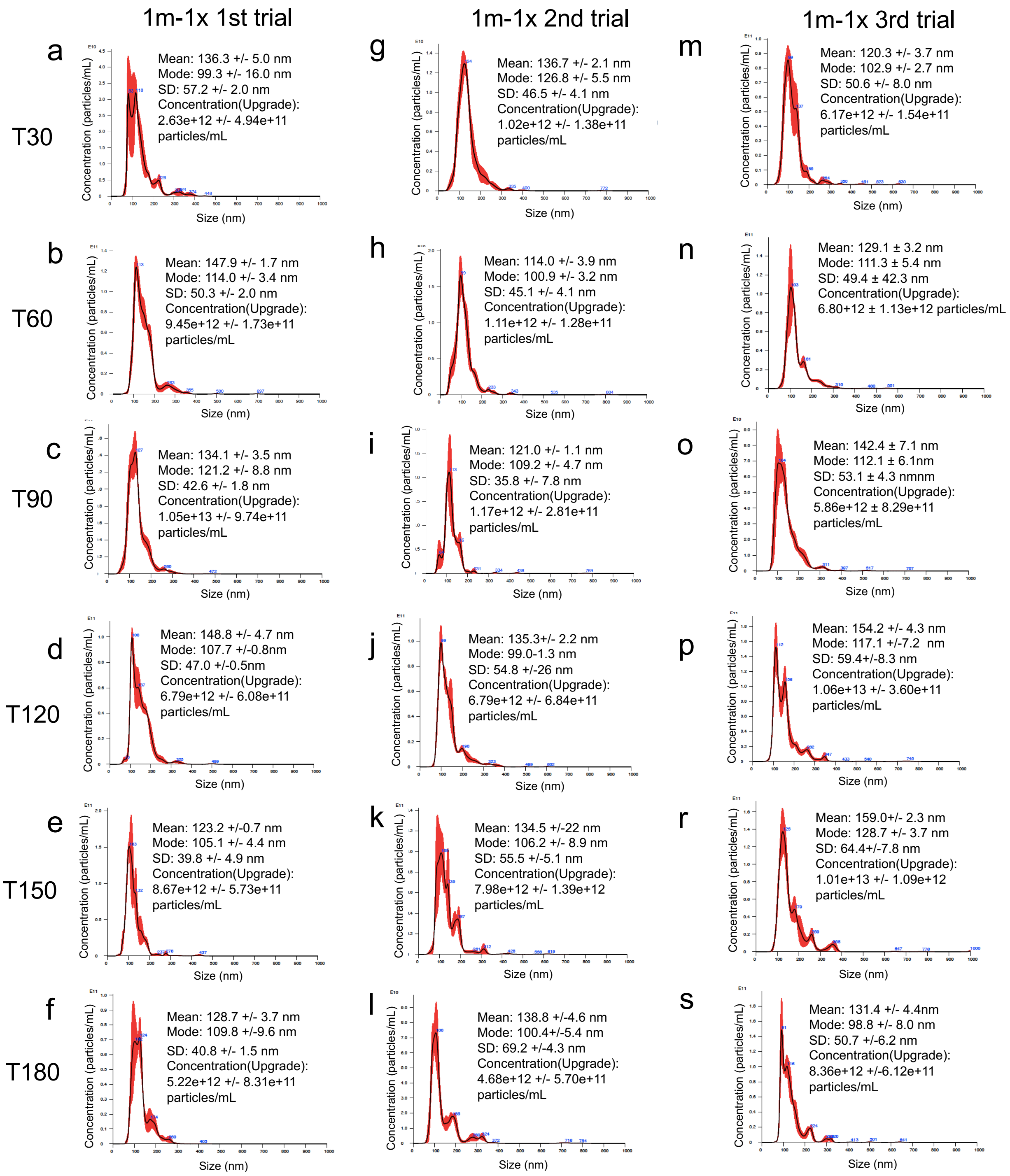

Supplementary Figure 5. The NTA results of BSA-imprinted nanoparticles collected at 30 min intervals with a flow rate of 1X and channel length of 1m were analyzed in three trials. Multiple trials of NTA analyses are presented as the 1st trial in (a-f), the 2nd trial in (g-l), and the 3rd trial in (m-s).

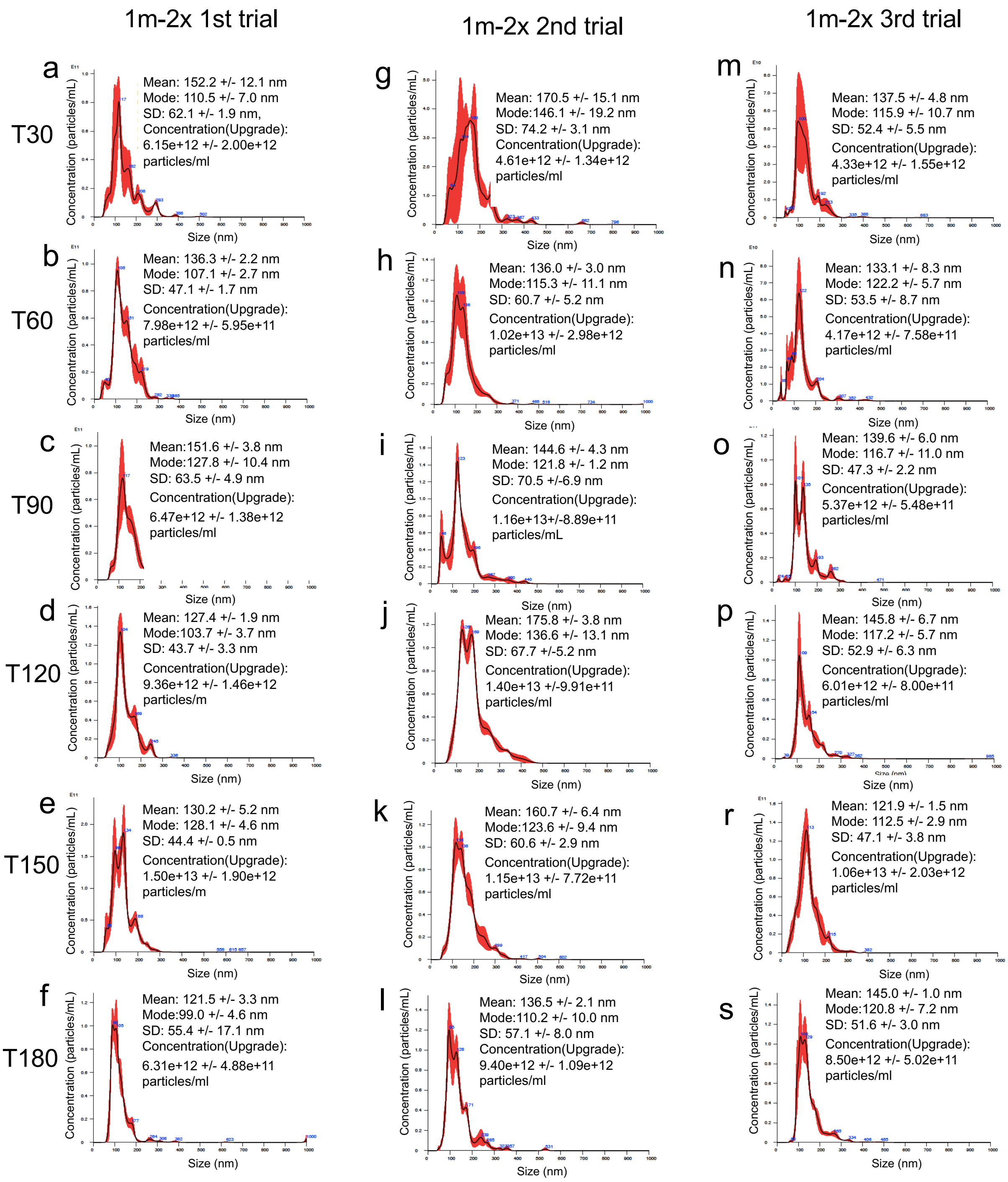

Supplementary Figure 6. The NTA results of BSA-imprinted nanoparticles collected at 30 min intervals with a flow rate of 2X and channel length of 1m were analyzed in three trials. Multiple trials of NTA analyses are presented as the 1st trial in (a-f), the 2nd trial in (g-l), and the 3rd trial in (m-s).

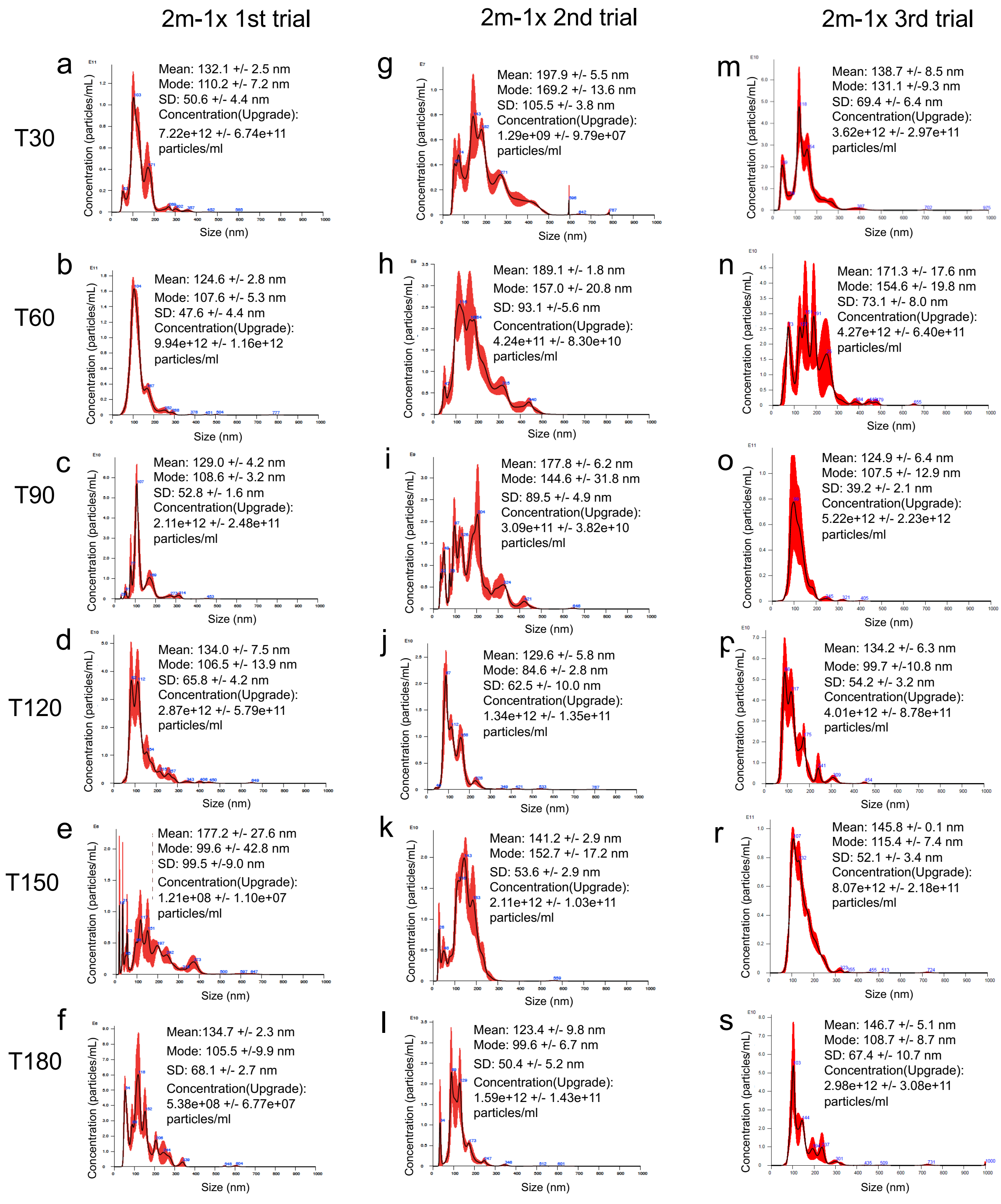

Supplementary Figure 7. The NTA results of BSA-imprinted nanoparticles collected at 30 min intervals with a flow rate of 1X and channel length of 2m were analyzed in three trials. Multiple trials of NTA analyses are presented as the 1st trial in (a-f), the 2nd trial in (g-l), and the 3rd trial in (m-s).

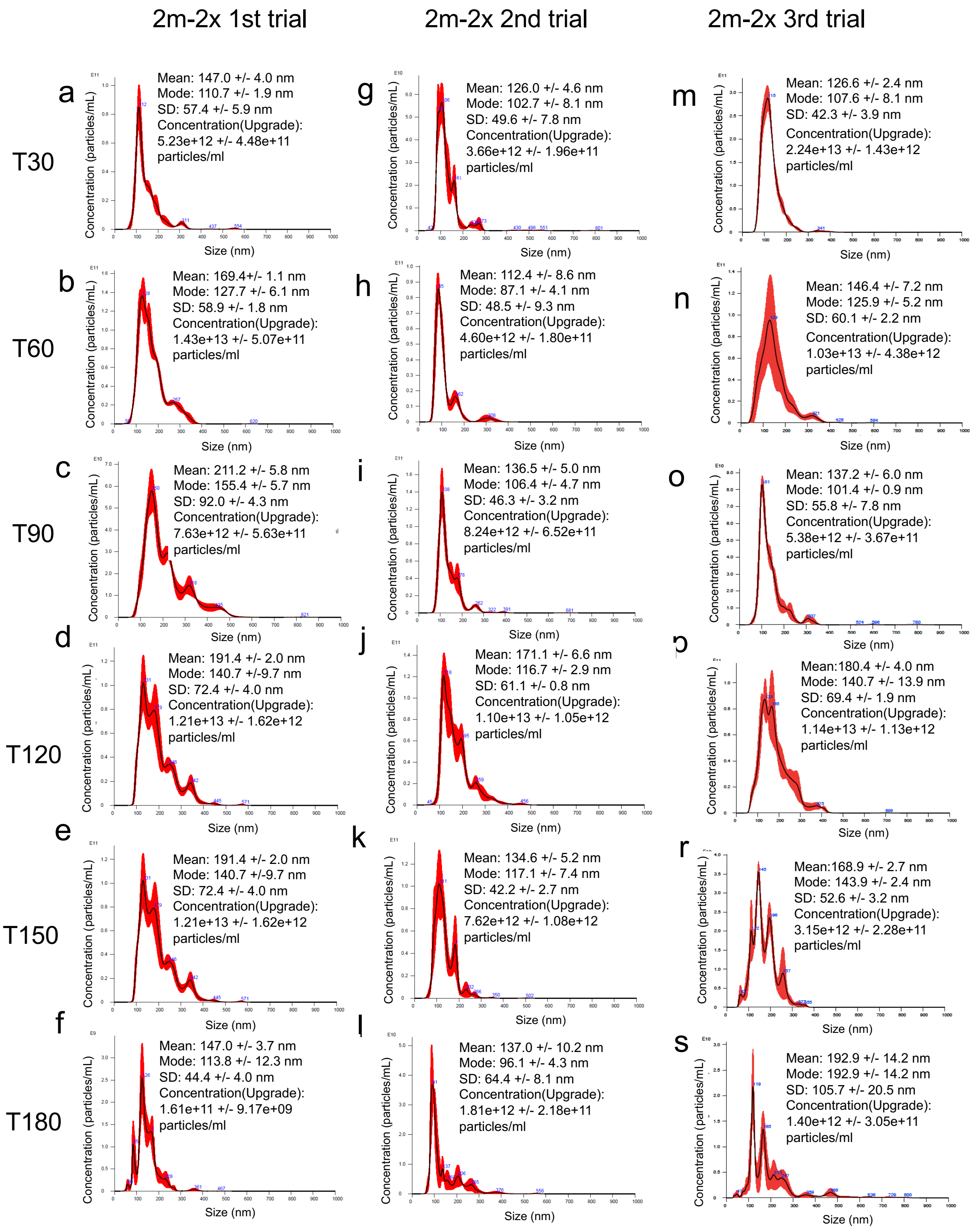

Supplementary Figure 8. The NTA results of BSA-imprinted nanoparticles collected at 30 min intervals with a flow rate of 2X and channel length of 2m were analyzed in three trials. Multiple trials of NTA analyses are presented as the 1st trial in (a-f), the 2nd trial in (g-l), and the 3rd trial in (m-s).

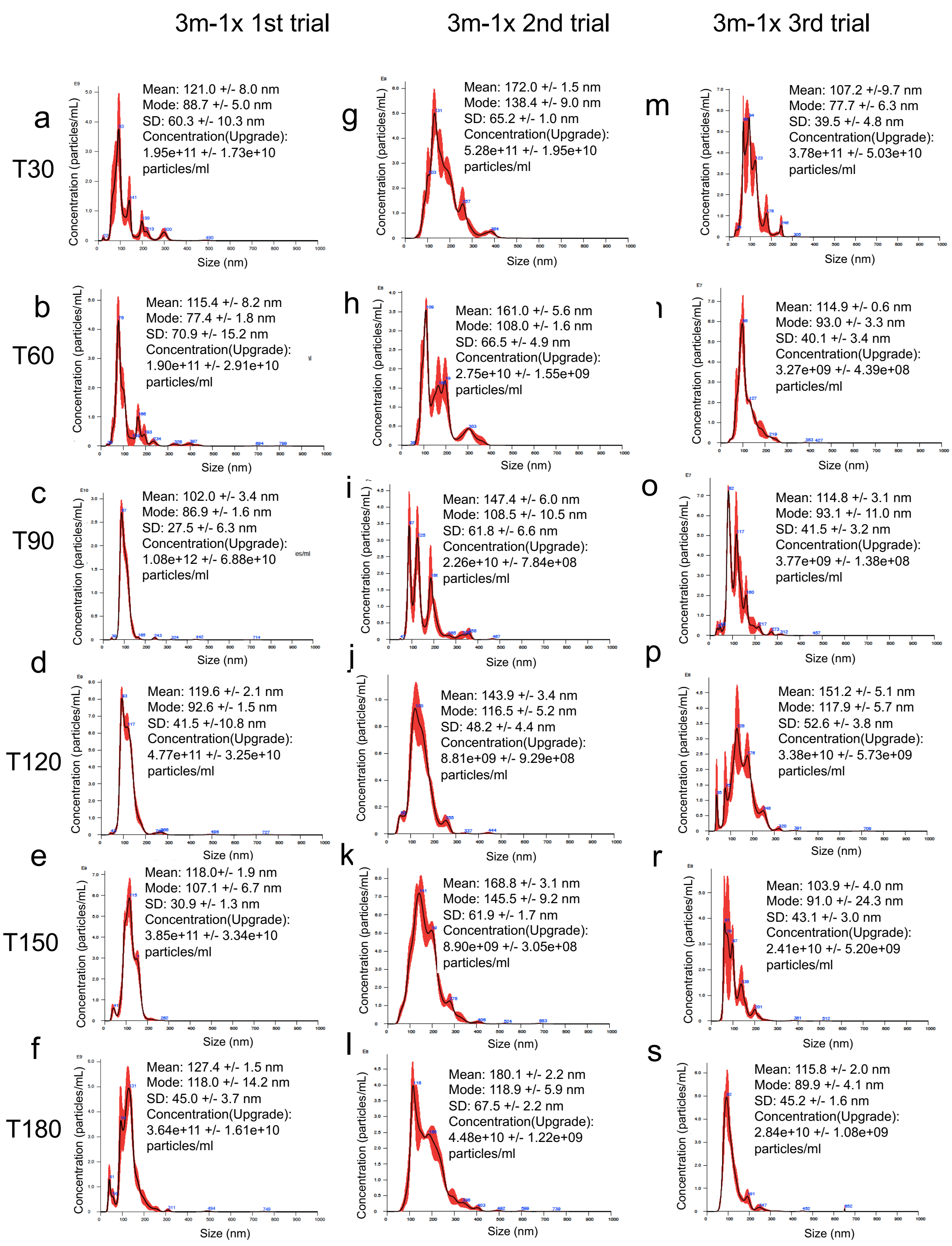

Supplementary Figure 9. The NTA results of BSA-imprinted nanoparticles collected at 30 min intervals with a flow rate of 1X and channel length of 3m were analyzed in three trials. Multiple trials of NTA analyses are presented as the 1st trial in (a-f), the 2nd trial in (g-l), and the 3rd trial in (m-s).

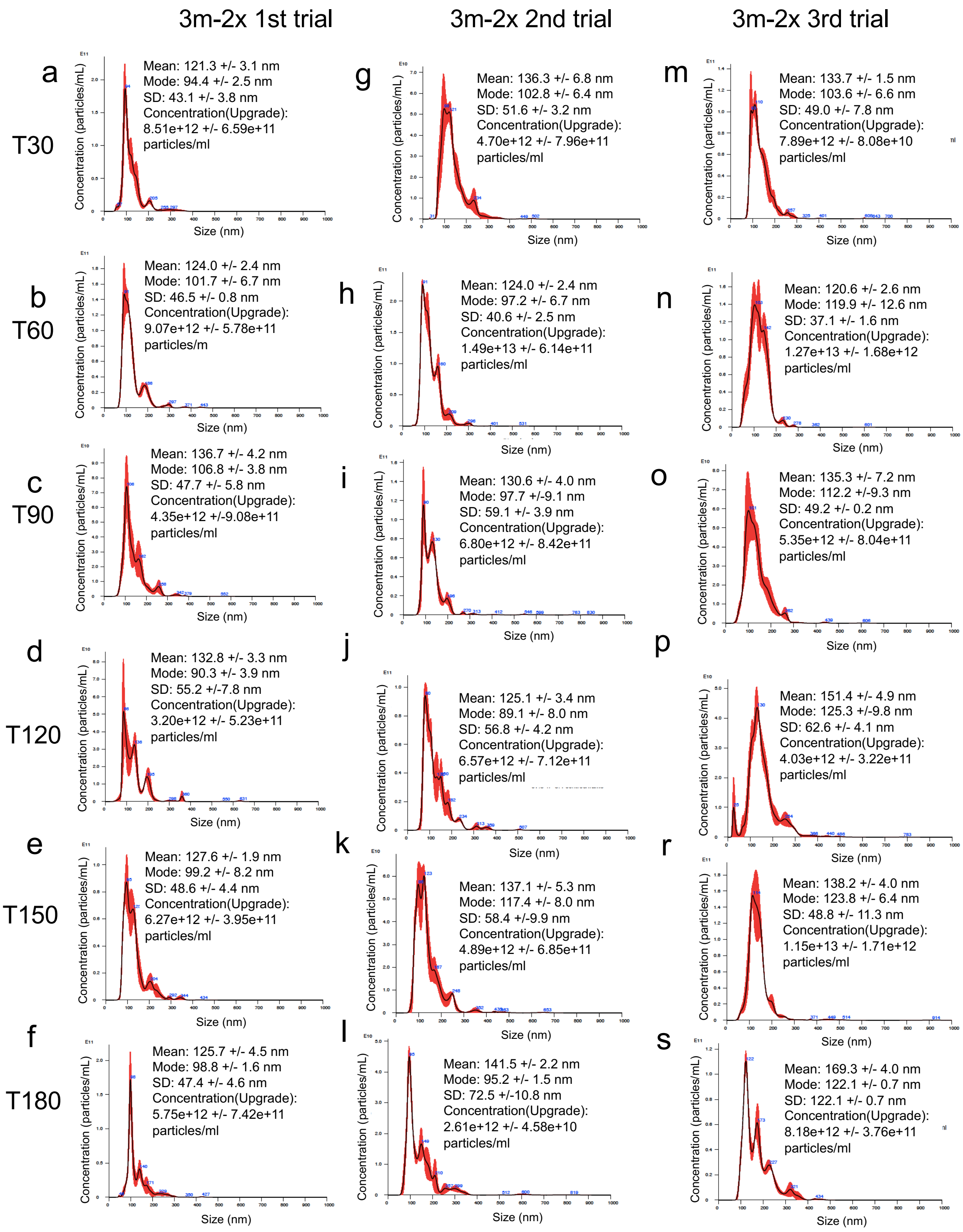

Supplementary Figure 10. The NTA results of BSA-imprinted nanoparticles collected at 30 min intervals with a flow rate of 2X and channel length of 3m were analyzed in three trials. Multiple trials of NTA analyses are presented as the 1st trial in (a-f), the 2nd trial in (g-l), and the 3rd trial in (m-s).

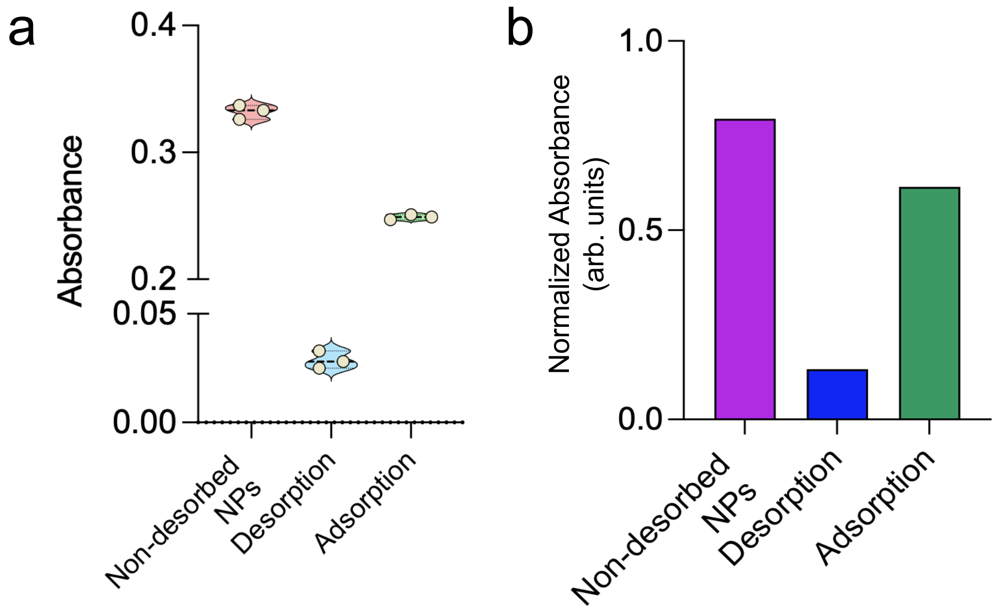

Supplementary Figure 11. (a) Absorbance measurements are demonstrated for non-desorbed, desorbed, and re-adsorbed nanoparticles. (b) Normalized absorbance data are calculated for non-desorbed, desorbed, and re-adsorbed nanoparticles.

Supplementary Table 1. The values of free energy resulted from the docking studies.

#Position	ΔG (Kcal/mol)
**#1**	-5.37
**#2**	-5.69
**#3**	-5.76
**#4**	-5.53
**#5**	-5.55
**#6**	-5.84
**#7**	-5.57
**#8**	-5.63
**#9**	-5.57
**#10**	-5.72

Supplementary Table 2. The interaction energy of MA, HEMA and MA-HEMA dimer with BSA.

Structure	Interaction Energy (Kcal/mol)
**MA-BSA**	-16.1921
**HEMA-BSA**	-16.0845
**MA-HEMA-BSA**	-35.6247

Supplementary Table 3. The cumulative dry-weight of MIPs collected at different time intervals.

Time (min)	Weight (mg) Micro-reactor	Weight (mg) Conventional
**30**	47.2	31.2
**60**	97.6	61.7
**90**	138.6	92.3
**120**	186.7	124.1
**150**	224.8	155.5
**180**	263.8	187

Supplementary References

1. Saylan, Y., Erdem, Ö., Cihangir, N. & Denizli, A. Detecting Fingerprints of Waterborne Bacteria on a Sensor. *Chemosensors* **7**, 33 (2019).
2. Altintas, Z. *et al.* Detection of Waterborne Viruses Using High Affinity Molecularly Imprinted Polymers. *Anal. Chem.* **87**, 6801–7 (2015).
3. Erdem, Ö., Cihangir, N., Saylan, Y. & Denizli, A. Comparison of molecularly imprinted plasmonic nanosensor performances for bacteriophage detection. *New J. Chem.* **44**, 17654–17663 (2020).
4. Information, (NCBI) National Center for Biotechnology. 2-Hydroxyethyl methacrylate | C6H10O3 - PubChem. *Pubchem* [https://pubchem.ncbi.nlm.nih.gov/compound/2-Hydroxyethyl-methacrylate#section=Solubility] (2022).
5. (NCBI) National Center for Biotechnology Information. Methacrylic acid | C4H6O2 - PubChem. *PubChem* [https://pubchem.ncbi.nlm.nih.gov/compound/Methacrylic-acid] (2022).
6. Pettersen, E. F. *et al.* UCSF Chimera—a visualization system for exploratory research and analysis. *J. Comput. Chem.* **25**, 1605–1612 (2004).
7. Dennington, R., Keith, T. A. & Millam, J. M. GaussView, version 6.0. 16. *Semichem Inc Shawnee Mission KS* (2016).
8. Jo, S., Kim, T., Iyer, V. G. & Im, W. CHARMM-GUI: a web-based graphical user interface for CHARMM. *J. Comput. Chem.* **29**, 1859–1865 (2008).
9. Frisch, M. J. & Clemente, F. R. Gaussian 09, revision a. 01, mj frisch, gw trucks, hb schlegel, ge scuseria, ma robb, jr cheeseman, g. *Scalmani, V. Barone, B. Mennucci, GA Petersson, H. Nakatsuji, M. Caricato, X. Li, HP Hratchian, AF Izmaylov, J. Bloino, G. Zhe* 20–44 (2009).
10. Stephens, P. J., Devlin, F. J., Chabalowski, C. F. & Frisch, M. J. Ab initio calculation of vibrational absorption and circular dichroism spectra using density functional force fields. *J. Phys. Chem.* **98**, 11623–11627 (1994).
11. Yan, Y., Zhang, D., Zhou, P., Li, B. & Huang, S.-Y. HDOCK: a web server for protein–protein and protein–DNA/RNA docking based on a hybrid strategy. *Nucleic Acids Res.* **45**, W365–W373 (2017).
12. Grosdidier, A., Zoete, V. & Michielin, O. SwissDock, a protein-small molecule docking web service based on EADock DSS. *Nucleic Acids Res* **39**, (2011).
13. Phillips, J. C. *et al.* Scalable molecular dynamics on CPU and GPU architectures with NAMD. *J. Chem. Phys.* **153**, 44130 (2020).
14. Humphrey, W., Dalke, A. & Schulten, K. VMD: visual molecular dynamics. *J. Mol. Graph.* **14**, 33–38 (1996).
15. Huang, J. & MacKerell Jr, A. D. CHARMM36 all-atom additive protein force field: Validation based on comparison to NMR data. *J. Comput. Chem.* **34**, 2135–2145 (2013).
16. Jorgensen, W. L., Chandrasekhar, J., Madura, J. D., Impey, R. W. & Klein, M. L. Comparison of simple potential functions for simulating liquid water. *J. Chem. Phys.* **79**, 926–935 (1983).
17. Darden, T., York, D. & Pedersen, L. Particle mesh Ewald: An N· log (N) method for Ewald sums in large systems. *J. Chem. Phys.* **98**, 10089–10092 (1993).
18. Inci, F. Benchmarking a Microfluidic-Based Filtration for Isolating Biological Particles. *Langmuir* **38**, 1897–1909 (2022).

19. Inci, F. Bioinspired Material-Integrated Sensors for Improving Nanoplasmonic Characteristics. *Hacettepe J. Biol. Chem.* **50**, 193–204 (2022).
20. Çelik, O., Saylan, Y., Göktürk, I., Yılmaz, F. & Denizli, A. A surface plasmon resonance sensor with synthetic receptors decorated on graphene oxide for selective detection of benzylpenicillin. *Talanta* **253**, 123939 (2023).
21. Lu, W., Wang, S., Liu, R., Guan, Y. & Zhang, Y. Human serum albumin-imprinted polymers with high capacity and selectivity for abundant protein depletion. *Acta Biomater.* **126**, 249–258 (2021).
